# Assessment of nutritional status in the elderly: a proposed function-driven model

**DOI:** 10.29219/fnr.v62.1366

**Published:** 2018-04-17

**Authors:** Stina Engelheart, Robert Brummer

**Affiliations:** School of Medical Sciences, Örebro University, Örebro, Sweden

**Keywords:** nutritional status, nutritional assessment, elderly people, comprehensive geriatric assessment

## Abstract

**Background:**

There is no accepted or standardized definition of ‘malnutrition’. Hence, there is also no definition of what constitutes an adequate nutritional status. In elderly people, assessment of nutritional status is complex and is complicated by multi-morbidity and disabilities combined with nutrition-related problems, such as dysphagia, decreased appetite, fatigue, and muscle weakness.

**Objective:**

We propose a nutritional status model that presents nutritional status from a comprehensive functional perspective. This model visualizes the complexity of the nutritional status in elderly people.

**Design and results:**

The presented model could be interpreted as the nutritional status is conditional to a person’s optimal function or situation. Another way of looking at it might be that a person’s nutritional status affects his or her optimal situation. The proposed model includes four domains: (1) physical function and capacity; (2) health and somatic disorders; (3) food and nutrition; and (4) cognitive, affective, and sensory function. Each domain has a major impact on nutritional status, which in turn has a major impact on the outcome of each domain.

**Conclusions:**

Nutritional status is a multifaceted concept and there exist several knowledge gaps in the diagnosis, prevention, and optimization of treatment of inadequate nutritional status in elderly people. The nutritional status model may be useful in nutritional assessment research, as well as in the clinical setting.

The prevalence of malnutrition is reported to be 18–30% in different populations of elderly people in need of health care services (1–6). As yet, there is no established or accepted definition of ‘malnutrition’ although several definitions have been used in the scientific literature (3, 7, 8) and several proposals have been presented (9–12). Hence, there is also no definition of what constitutes an adequate nutritional status.

This article will not attempt to define malnutrition but will elaborate on nutritional status, as a condition, from a comprehensive functional perspective. Impaired nutritional status (as in malnutrition) may not itself be a subjective problem or discomfort, unless it affects the persons’ capacities or contributes to their impairments or disease progression. We propose a function-driven nutritional status model in order to visualize the diversity of the situation and also to analyze and discuss nutritional status.

The proposed nutritional status model, as well as the associated nutritional assessment, is developed from questions concerning how nutritional status affects, and is affected by, health or disease. In a young or adult population, the importance of an adequate nutritional status in supporting a long and healthy life is well established. But what about older people who are already in need of health care and social services? Is the aim still to lead a long and, depending on individual circumstances, relatively healthy life? Or is the aim to enable them to live an independent life? And how does the approach to nutritional status in old age affect practice in health care? Populations in geriatric care are heterogeneous, which further complicates the application of scientific research to individuals’ needs in health care.

## Need for personalized nutritional care

There is a need for an effective, personalized, and scientifically based model for the assessment and evaluation of nutritional status in old people. Today, most countries and communities are facing a geriatric challenge (13), with an increasing proportion of older people in the population (14). A geriatric nutritional assessment is complicated by multi-morbidity, injuries, and disabilities in combination with nutrition-related problems such as dysphagia, decreased appetite, fatigue, and muscle weakness. Old age is the most dominant risk factor for acute and chronic disease, as well as reduced physical, cognitive, affective, and social function. This functional decline may be the main reason for high risk of malnutrition (8, 15, 16), but the risk of malnutrition increases even further in the case of multi-morbidity, and such disease-related malnutrition is common in old people (17). The increased risk of malnutrition found in research may, however, be due to the method used for nutritional assessment, as some methods are based on parameters such as the number of drugs used, living arrangements (e.g. living at home vs. living in the nursing home), and diagnoses (e.g. dementia), indicating an increased risk of malnutrition according to the screening method. A minimum age, or definition of elderly people, for the proposed approach on nutritional status is not defined, as each individual situation has to be taken into concern. The concept of ‘frailty’ has been proposed to make a distinction between biological and chronological ages and is therefore applicable in this proposed model as it highlights the challenges of geriatric nutrition. Nutritional assessments require knowledge, qualified personnel, and scientifically based methods to evaluate and meet the nutritional needs of people at old age.

**Fig. 1:**
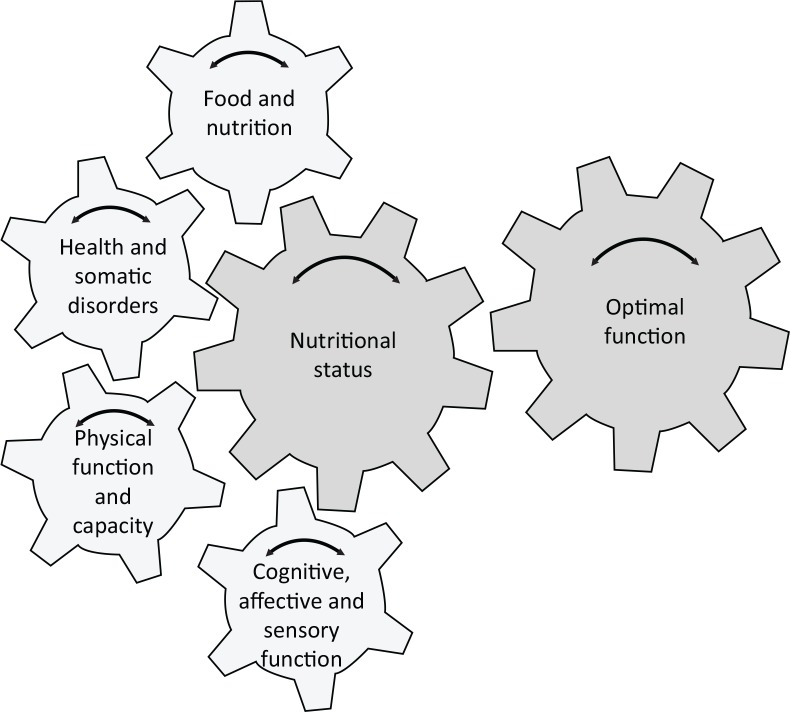
Individualized model for assessment of nutritional status, including an obligatory examination of each of four domains, namely, (1) physical function and capacity; (2) health and somatic disorders; (3) food and nutrition; and (4) cognitive, affective, and sensory function. Each domain contributes to nutritional status. Optimal function (defined for each person) is the most important factor in analyzing nutritional status and at the same time is the overall goal of any actions and treatment.

A comprehensive perspective is needed to adequately assess and interpret nutritional status in elderly people, as visualized in the proposed model for assessment of nutritional status (Fig. 1). The model takes account of the heterogeneity of the elderly population, with various symptoms, disorders, and treatments affecting their nutritional status. Nutritional research, as well as the clinical methodology of nutritional assessment, has to explore associations between nutritional status and its predictors, exposures, and outcomes. In clinical practice and also in research we need a personalized approach, taking into account the heterogeneity of the population and the complex nature of nutritional status (18). Too often research including nutritional assessments ignores the complexity of nutritional status in elderly people and uses a single parameter such as low body mass index or low energy intake, or else it is based on simple screening methods such as the Mini Nutritional Assessment or Subjective Global Assessment. Also, most instruments aim to evaluate the presence of malnutrition rather than to adequately assess nutritional status.

In the proposed nutritional status model, the goal for actions and treatment in the clinical setting is to identify and achieve optimal function (and the optimal situation) for each person or patient. The model describes the complex interaction between four domains contributing to the overall goal – the optimal function.

## Aim of the nutritional status model

The proposed model aims to visualize the interplay between the main components of nutritional status from an aging perspective. It is intended to be used in nutritional assessments in research as well as in clinical settings.

The four domains included in the proposed model of nutritional status were identified by two background questions: ‘What constitutes adequate nutritional status in old people?’ and ‘How do health care professionals perceive nutritional status in elderly people?’ The model should be applied flexibly, as the domains are interrelated and these interrelations are individually specific. The model may also require that in specific cases domains should be added or rebuilt.

The complexity of nutrition is highlighted within this research, as food has nutritional, social, biomedical, and functional implications. In the proposed model, we use four domains, overall categories or blocks in a comprehensive framework, to facilitate a fruitful discussion as part of the process of assessing nutritional status.

1. Physical function and capacity: comprising muscle strength, activities of daily living (ADL) functioning, physical activity, body composition, etc.
2. Health and somatic disorders: comprising prescribed pharmaceuticals, physical symptoms, current diseases, health-related quality of life, inflammation, etc.
3. Food and nutrition: comprising intake of energy and nutrients, mealtime habits, fluid intake, dietary patterns, etc.
4. Cognitive, affective, and sensory function: comprising cognitive decline, depression, mood, sense of taste and smell, etc.

In practical use, the model encourages transdisciplinary competence. No specific speciality has precedence of any area or domain, and no specific area or domain has precedence over any other area or domain, in the model.

The model could be interpreted as indicating that nutritional status is conditional to a person’s optimal function (as defined by themselves), where each of the four domains contributes to the final goal (of optimal function). Also, the model could be interpreted as showing that nutritional status affects each domain and, consequently, the individual’s optimal function. Each domain has a great impact on nutritional status, which has a great impact on the outcome of the domains, as explained below. In each individual case, each domain is more or less important for achieving optimal function, as the optimal function is defined by each person and each particular situation or setting.

In the following, the four domains are described and presented from this perspective.

### Physical function and capacity

In the framework of this article, ‘physical function’ mainly comprises muscle, cardiovascular, and pulmonary function. Physical function is not necessarily related to physical capacity. The muscle function of leg muscles (measured using leg press) may be adequate, but when it comes to the capacity of performing household chores, the physical (i.e. muscle) function (e.g. strong legs) has to be transformed to physical capacity (e.g. walking, standing, bending, and keeping the balance). In general, loss of muscle mass is associated with loss of functional capacity and also with the risk of developing chronic metabolic disease (19).

Change in body weight is often used as a primary outcome measure in nutritional interventions in the elderly, in research, as well as in clinical practice. As an isolated biomarker, the individual’s physical capacity will probably matter more than body weight. The ability to perform ADL is highly relevant in this context, as loss of muscle cell mass is related to loss of ADL function (20), and malnutrition is correlated with dependence on other people for ADLs (21). Nutritional interventions (dietary advice and nutritional supplementation) with the goal to improve ADL functions are most useful for people at risk of malnutrition (22).

Body composition is strongly related to nutritional status. Body fat mass and fat-free mass are associated with physical ability, morbidity, and mortality (23, 24)*.* Body composition changes in old age, even in individuals with a stable body weight, and is characterized by increased fat mass and reduced fat-free mass (25). This change, probably due to hormonal changes, inadequate nutritional intake, increased morbidity, and less physical activity and exercise, among other reasons, causes sarcopenia and impaired physical function (7, 26).

Older people, especially those with multi-morbidity, have increased levels of systemic inflammatory markers, such as C-reactive protein (CRP) (26), and chronic inflammation also denoted as ‘inflammaging’. Increased levels of inflammatory activity impair the anabolic processes in the body, as an anabolic block (27). The inflammatory condition may also decrease the intake of energy through loss of appetite, a condition also called ‘anorexia of ageing’ (28). Nutritional interventions in such a catabolic state are complicated and should not focus merely on achieving a positive energy balance, as this will result in increased body weight, meaning predominantly increased body fat. This may, in turn, stimulate the systemic inflammatory activity and hamper the anabolic processes even further (28).

In summary, physical function and capacity affect nutritional status in a bidirectional fashion. Changes in body weight as an indicator of a person’s nutritional status have to be measured in terms of various body function indicators, as a complement. Physical function and capacity may be measured as muscle strength, ADL function, physical activity, body composition, etc.

### Health and somatic disorders

Disease may negatively affect appetite, which can, in turn, lead to impairment of nutritional status and functional performance. As described previously in this article, old age is associated with chronic systemic inflammation (inflammaging), which substantially affects morbidity and mortality (29). Physical exercise has been discussed as preventive action (30), but its effect has not been clearly proven. The presence of this systemic inflammatory activity also disqualifies the use of serum albumin concentrations as a valid indicator of nutritional status. Because of its characteristic as a negative acute phase protein, it reflects inflammatory status rather than indicating adequate protein intake in particular, or nutritional status in general (31).

Acute or somatic disorders, and their treatment and the resulting functional impairments, may negatively affect the ability to ingest and/or digest a meal, as well as to absorb macro- and micronutrients, hence negatively affecting nutritional status. A poor nutritional status also impairs the immune function, increasing the risk of disease and contributing to a negative trend. Disease and multi-morbidity have traditionally been considered as a confounder (or just ignored) in the research on nutritional status and malnutrition. However, in the model presented here, this is an essential part of and contributor to nutritional status. Classification of disease status could not only be achieved by diagnosis or a combination of diagnoses, but also the number or category of pharmaceuticals can be an indicator of disease status.

The presence of physical or psychological symptoms, due to disorders, may affect not only dietary intake but also other components of lifestyle, such as physical activity and social interactions. Conversely, poor nutritional status may have an impact on physical capacity and social interaction and consequently will affect the quality of life.

In summary, the presence of disease and multi-morbidity, and the inflammation and symptoms they may cause, closely affect the nutritional status in a bidirectional fashion. The domain of health and somatic disorders may be measured as prescribed or used pharmaceuticals, physical symptoms, current diseases, health-related quality of life, inflammation, etc.

### Food and nutrition

Old age *per se* does not cause reduced dietary intake. However, if functions required for habitual activities (such as shopping, cooking, and eating) are compromised due to disease or reduced capacity, then the intake of energy and nutrients will be decreased (32–35). The changes in food habits, in combination with the ongoing disease, challenge the health practitioners to provide individualized care and achieve a comprehensive view of the person’s nutritional status.

The Nordic Nutrition Recommendations (NNR) (36) include dietary reference values for nutrients, foods, food patterns, physical activity, and sustainable food, with the aim to help prevent illnesses and chronic diseases. The reference values are adapted to different age groups, from infants to older adults, in good health. The use of reference values in frail elderly people, or those at immediate risk of frailty or malnutrition, is complicated and, hence, determining adequate nutritional intake on an individual basis in these elderly individuals is cumbersome and not evidence based. Therefore, more research in needed for this specific group of people. On an individual level, the care needs to be based on, among others, a comprehensive examination of energy need, body composition, physical function, and biomarkers.

The intake of fluids is rarely included in the analysis of dietary intake, although it is an essential contributor to optimal metabolic function and nutritional status. An impaired ability to achieve essential hydration status in combination with decreased fluid intake is common in old age (36, 37), and overt dehydration has been reported in old people in need of health and social care (38–40). However, the importance of fluid intake is probably underestimated in clinical practice (41). Low fluid intake is not synonymous with dehydration, as the risk of dehydration is also affected by the presence of diseases, and their treatment, as well as by the person’s general physical condition. Unfortunately, the impact of dehydration and insufficient fluid intake in old age is insufficiently studied, although confusion and cognitive impairment have been reported as symptoms (42, 43). Impaired cognitive performance may occur with dehydration matching only 2% of the person’s body weight (44), and older people may reach this level of dehydration earlier than younger people, as body water volume decreases with age (45). The NNR (36) presents a guiding value of water and fluid intake (in addition to water from foods) at 1–1.5 L/day for adults. There is no specific recommendation for older people, but it is concluded that elderly people should have a broader safety margin due to less capacity to concentrate urine and often impaired feelings of thirst.

Food and nutrition is probably the domain most obviously associated with nutritional status, but it is complex as it comprises aspects such as adequate intake of macro- and micronutrients, dietary patterns, mealtime situation, mealtime habits, surrounding environment, and social interaction during meals. Food intake may be perceived as a pleasant event, but can also be a medical treatment, as well as a necessity for survival. Hence, the solution to an individual nutritional problem needs to be more than a recommendation of a specific dietary intake (8), and nutritional intervention studies should include a functional perspective in the nutritional assessments or outcomes.

In summary, food and nutrition, as the most obvious of the domains included, affect nutritional status in a bidirectional fashion. The food and nutrition domain should be analyzed from a broader perspective.

### Cognitive, affective, and sensory function

Adequate cognitive function is crucial for most activities in daily living, including planning and preparing meals, food intake, physical exercise, and other factors contributing to adequate nutritional status. In the care of people with dementia, the importance of creating a dining environment based on each and every person has been emphasized (46), as the physical environment has a major impact on the food and meal experience and, hence, the person’s nutritional status.

The definition of ‘cognitive function’ may comprise mood, regulation of anxiety, concentration, memory, and motivation or initiative. Most available scientific reports on the interaction between nutritional status and cognitive function deal with the hydration issue, or with the impact of dementia. Malnutrition is more common in people with dementia (47), with difficulties handling mealtimes during the progression of the disease (48). The identified increase in risk may also be due to the methods used for nutritional assessment.

An association between impaired nutritional status and depression has been observed, but the causal relationship is complex and it can be questioned whether depression is the cause or consequence of impaired nutritional status (49).

Impairment of olfactory function worsens with age, and the prevalence is higher in malnourished and multi-morbid people (50). This may negatively affect dietary intake, and it may cause specific micronutrient deficiencies that may, in turn, deteriorate olfactory function. However, the association between malnutrition and olfactory function has not been widely investigated and can be questioned (51).

In summary, cognitive, affective, and sensory functions affect nutritional status in a bidirectional fashion. To assess this domain, measurements of cognitive function or decline, depression, mood, and sensory function such as taste and smell can be used.

## Conclusion

A model for assessing nutritional status is presented. We argue that nutritional status is a multi-faceted concept and the presented model highlights the complexity. Several knowledge gaps exist in each domain, leading to uncertainty and lack of evidence on how to diagnose, prevent, and optimize nutritional status in an individual and personalized setting.

The proposed nutritional status model has been used in a research setting but not in regular clinical setting. The model should, therefore, be implemented in various settings in order to generate experience. In research setting, it has supported the understanding of the complex role of nutrition in the health and well-being of the elderly, at a group level as well as at an individual level, supporting comprehensive geriatric assessment.

## References

[1] Johansson Y, Bachrach-Lindstrom M, Carstensen J, Ek AC Malnutrition in a home-living older population: prevalence, incidence and risk factors. A prospective study. J Clin Nurs 2009; 18(9): 1354–64. 19077017 10.1111/j.1365-2702.2008.02552.x

[2] Odlund Olin A, Koochek A, Cederholm T, Ljungqvist O Minimal effect on energy intake by additional evening meal for frail elderly service flat residents – a pilot study. J Nutr Health Aging 2008; 12(5): 295–301. 18443710 10.1007/BF02982658 PMC12892652

[3] Meijers JM, van Bokhorst-de van der Schueren MA, Schols JM, Soeters PB, Halfens RJ Defining malnutrition: mission or mission impossible? Nutrition 2010; 26(4): 432–40. 19954929 10.1016/j.nut.2009.06.012

[4] Torma J, Winblad U, Cederholm T, Saletti A Does undernutrition still prevail among nursing home residents? Clin Nutr 2013; 32(4): 562–8. 23137706 10.1016/j.clnu.2012.10.007

[5] Serrano-Urrea R, Garcia-Meseguer MJ Malnutrition in an elderly population without cognitive impairment living in nursing homes in SpaIn: study of prevalence using the Mini Nutritional Assessment test. Gerontology 2013; 59(6): 490–8. 23949114 10.1159/000351763

[6] van Nie-Visser NC, Meijers J, Schols J, Lohrmann C, Bartholomeyczik S, Spreeuwenberg M, et al. Which characteristics of nursing home residents influence differences in malnutrition prevalence? An international comparison of The Netherlands, Germany and Austria. Br J Nutr 2014; 111(6): 1129–36. 24246053 10.1017/S0007114513003541

[7] Cruz-Jentoft AJ, Baeyens JP, Bauer JM, Boirie Y, Cederholm T, Landi F, et al. Sarcopenia: European consensus on definition and diagnosis: report of the European Working Group on Sarcopenia in Older People. Age Ageing 2010; 39(4): 412–23. 20392703 10.1093/ageing/afq034 PMC2886201

[8] Evans WJ, Morley JE, Argiles J, Bales C, Baracos V, Guttridge D, et al. Cachexia: a new definition. Clin Nutr 2008; 27(6): 793–9. 18718696 10.1016/j.clnu.2008.06.013

[9] White JV, Guenter P, Jensen G, Malone A, Schofield M, Academy of Nutrition and Dietetics Malnutrition Work Group, et al Consensus statement of the Academy of Nutrition and Dietetics/American Society for Parenteral and Enteral Nutrition: characteristics recommended for the identification and documentation of adult malnutrition (undernutrition). J Acad Nutr Diet 2012; 112(5): 730–8. 22709779 10.1016/j.jand.2012.03.012

[10] van Bokhorst-de van der Schueren MA, Guaitoli PR, Jansma EP, de Vet HC A systematic review of malnutrition screening tools for the nursing home setting. J Am Med Dir Assoc 2014; 15(3): 171–84. 24290910 10.1016/j.jamda.2013.10.006

[11] Cederholm T, Jensen GL To create a consensus on malnutrition diagnostic criteria: a report from the Global Leadership Initiative on Malnutrition (GLIM) meeting at the ESPEN Congress 2016. Clin Nutr 2017; 36(1): 7–10. 28034565 10.1016/j.clnu.2016.12.001

[12] Fischer M, JeVenn A, Hipskind P Evaluation of muscle and fat loss as diagnostic criteria for malnutrition. Nutr Clin Pract 2015; 30(2): 239–48. 25753808 10.1177/0884533615573053

[13] Christensen K, Doblhammer G, Rau R, Vaupel JW Ageing populations: the challenges ahead. Lancet 2009; 374(9696): 1196–208. 19801098 10.1016/S0140-6736(09)61460-4 PMC2810516

[14] European Commission Eurostat. Your key to European statistics., 2014.

[15] Saragat B, Buffa R, Mereu E, Succa V, Cabras S, Mereu RM, et al. Nutritional and psycho-functional status in elderly patients with Alzheimer’s disease. J Nutr Health Aging 2012; 16(3): 231–6. 22456778 10.1007/s12603-011-0347-3 PMC12878098

[16] Donini LM, Savina C, Rosano A, Cannella C Systematic review of nutritional status evaluation and screening tools in the elderly. J Nutr Health Aging 2007; 11(5): 421–32. 17657364

[17] Marengoni A, Winblad B, Karp A, Fratiglioni L Prevalence of chronic diseases and multimorbidity among the elderly population in Sweden. Am J Public Health 2008; 98(7): 1198–200. 18511722 10.2105/AJPH.2007.121137 PMC2424077

[18] Engelheart S, Akner G Dietary intake of energy, nutrients and water in elderly people living at home or in nursing home. J Nutr Health Aging 2015; 19(3): 265–72. 25732210 10.1007/s12603-015-0440-0 PMC12877514

[19] Koopman R, van Loon LJC Aging, exercise, and muscle protein metabolism. J Appl Physiol 2009; 106(6): 2040–8. 19131471 10.1152/japplphysiol.91551.2008

[20] Zuliani G, Romagnoni F, Volpato S, Soattin L, Leoci V, Bollini MC, et al. Nutritional parameters, body composition, and progression of disability in older disabled residents living in nursing homes. J Gerontol A Biol Sci Med Sci 2001; 56(4): M212–16. 11283193 10.1093/gerona/56.4.m212

[21] van Bokhorst-de van der Schueren MA, Lonterman-Monasch S, de Vries OJ, Danner SA, Kramer MH, Muller M Prevalence and determinants for malnutrition in geriatric outpatients. Clin Nutr 2013; 32(6): 1007–11. 23755842 10.1016/j.clnu.2013.05.007

[22] Persson M, Hytter-Landahl A, Brismar K, Cederholm T Nutritional supplementation and dietary advice in geriatric patients at risk of malnutrition. Clin Nutr 2007; 26(2): 216–24. 17275141 10.1016/j.clnu.2006.12.002

[23] French SA, Folsom AR, Jeffery RW, Williamson DF Prospective study of intentionality of weight loss and mortality in older women: the Iowa Women’s Health Study. Am J Epidemiol 1999; 149(6): 504–14. 10084239 10.1093/oxfordjournals.aje.a009844

[24] Allison DB, Zannolli R, Faith MS, Heo M, Pietrobelli A, VanItallie TB, et al. Weight loss increases and fat loss decreases all-cause mortality rate: results from two independent cohort studies. Int J Obes Relat Metab Disorders 1999; 23(6): 603–11. 10.1038/sj.ijo.0800875 10411233

[25] Beaufrère B, Morio B Fat and protein redistribution with aging: metabolic considerations. Eur J Clin Nutr 2000; 54(Suppl 3): S48–53. 11041075 10.1038/sj.ejcn.1601025

[26] Vasto S, Candore G, Balistreri CR, Caruso M, Caruso C, Colonna-Romano G, et al. Inflammatory networks in ageing, age-related diseases and longevity. Mech Ageing Dev 2007; 128(1): 83–91. 17118425 10.1016/j.mad.2006.11.015

[27] Franceschi C. Inflammaging as a major characteristic of old people: can it be prevented or cured? Nutr Rev 2007; 65(12 Pt 2): S173–6. 18240544 10.1111/j.1753-4887.2007.tb00358.x

[28] Fernandez-Real JM, Ferri MJ, Vendrell J, Ricart W Burden of infection and fat mass in healthy middle-aged men. Obesity 2007; 15(1): 245–52. 17228053 10.1038/oby.2007.541

[29] Franceschi C, Bonafe M, Valensin S, Olivieri F, De Luca M, Ottaviani E, et al. Inflamm-aging. An evolutionary perspective on immunosenescence. Ann N Y Acad Sci 2000; 908: 244–54. 10911963 10.1111/j.1749-6632.2000.tb06651.x

[30] de Araujo AL, Silva LC, Fernandes JR, Benard G Preventing or reversing immunosenescence: can exercise be an immunotherapy? Immunotherapy 2013; 5(8): 879–93. 23902557 10.2217/imt.13.77

[31] Sullivan DH, Johnson LE, Dennis RA, Roberson PK, Heif M, Garner KK, et al. The Interrelationships among albumin, nutrient intake, and inflammation in elderly recuperative care patients. J Nutr Health Aging 2011; 15(4): 311–15. 21437564 10.1007/s12603-010-0297-1 PMC12879678

[32] Lammes E, Akner G Repeated assessment of energy and nutrient intake in 52 nursing home residents. J Nutr Health Aging 2006; 10(3): 222–30. 16622584

[33] Suominem M, Laine T, Routasalo P, Pitkala KH, Rasanen L Nutrient content of served food, nutrient intake and nutritional status of residents with dementia in a Finnish nursing home. J Nutr Health Aging 2004; 8(4): 234–8. 15316587

[34] Akner G, Flöistrup H Individual assessment of intake of energy, nutrients and water in 54 elderly multidiseased nursing-home residents. J Nutr Health Aging 2003; 7(1): 1–12. 12679834

[35] Nowson CA, Sherwin AJ, McPhee JG, Wark JD, Flicker L Energy, protein, calcium, vitamin D and fibre intakes from meals in residential care establishments in Australia. Asia Pac J Clin Nutr 2003; 12(2): 172–7. 12810407

[36] Norden Nordic nutrition recommendations 2012. Integrating nutrition and physical activity. Copenhagen: Nordic Council of Ministers; 2014.

[37] Andreucci VE, Russo D, Cianciaruso B, Andreucci M Some sodium, potassium and water changes in the elderly and their treatment. Nephrol Dial Transplant 1996; 11(Suppl 9): 9–17. 10.1093/ndt/11.supp9.9 9050029

[38] Buffa R, Mereu RM, Putzu PF, Floris G, Marini E Bioelectrical impedance vector analysis detects low body cell mass and dehydration in patients with Alzheimer’s disease. J Nutr Health Aging 2010; 14(10): 823–7. 21125199 10.1007/s12603-010-0115-9 PMC12879604

[39] Armstrong-Esther CA, Browne KD, Armstrong-Esther DC, Sander L. The institutionalized elderly: dry to the bone! Int J Nurs Stud 1996; 33(6): 619–28. 8970859 10.1016/s0020-7489(96)00023-5

[40] Mentes JC A typology of oral hydration problems exhibited by frail nursing home residents. J Gerontol Nurs 2006; 32(1): 13–19; quiz 20–1. 10.3928/0098-9134-20060101-09 16475460

[41] Popkin BM, D’Anci KE, Rosenberg IH Water, hydration, and health. Nutr Rev 2010; 68(8): 439–58. 20646222 10.1111/j.1753-4887.2010.00304.x PMC2908954

[42] Benton D Dehydration influences mood and cognition: a plausible hypothesis? Nutrients 2011; 3(5): 555–73. 22254111 10.3390/nu3050555 PMC3257694

[43] Secher M, Ritz P Hydration and cognitive performance. J Nutr Health Aging 2012; 16(4): 325–9. 22499450 10.1007/s12603-012-0033-0 PMC12878011

[44] Grandjean AC, Grandjean NR Dehydration and cognitive performance. J Am Coll Nutr 2007; 26(5 Suppl): 549S–54S. 17921464 10.1080/07315724.2007.10719657

[45] Lieberman HR Hydration and cognition: a critical review and recommendations for future research. J Am Coll Nutr 2007; 26(5 Suppl): 555–61. 10.1080/07315724.2007.10719658 17921465

[46] Chaudhury H, Hung L, Badger M The role of physical environment in supporting person-centered dining in long-term care: a review of the literature. Am J Alzheimers Dis Other Demen 2013; 28(5): 491–500. 23687182 10.1177/1533317513488923 PMC10852766

[47] Odlund Olin A, Koochek A, Ljungqvist O, Cederholm T Nutritional status, well-being and functional ability in frail elderly service flat residents. Eur J Clin Nutr 2005; 59(2): 263–70. 15483631 10.1038/sj.ejcn.1602067

[48] Keller HH, Edward HG, Cook C Mealtime experiences of families with dementia. Am J Alzheimers Dis Other Demen 2007; 21(6): 431–8. 10.1177/1533317506294601 17267376

[49] Smoliner C, Norman K, Wagner K-H, Hartig W, Lochs H, Pirlich M Malnutrition and depression in the institutionalised elderly. Br J Nutr 2009; 102(11): 1663–7. 19622192 10.1017/S0007114509990900

[50] Toffanello ED, Inelmen EM, Imoscopi A, Perissinotto E, Coin A, Miotto F, et al. Taste loss in hospitalized multimorbid elderly subjects. Clin Interv Aging 2013; 8: 167–74. 23426191 10.2147/CIA.S37477 PMC3576013

[51] Smoliner C, Fischedick A, Sieber CC, Wirth R Olfactory function and malnutrition in geriatric patients. J Gerontol A Biol Sci Med Sci 2013; 68(12): 1582–8. 23833205 10.1093/gerona/glt085

